# Supplementary material for: Reviewing the potentials of MMOGs as research environments: A case study from the strategy game Travian
Source: PLoS One. 2023 Feb 15;18(2):e0281114. doi: 10.1371/journal.pone.0281114 (PMC9931136; doi:10.1371/journal.pone.0281114)
Supplement: Attachment — Submitted filename: Response to Reviewers.pdf [file pone.0281114.s003.pdf]

Bavarian School of Public Policy
Technical University of Munich

Hochschule für Politik München | Richard-Wagner-Straße 1 | 80333 München
Professor of Computational Social Science & Big Data

Munich, July 27, 2022

Journal PLOS ONE
Submission: Revised research article

Response to Reviewers

Dear Reviewers,

Thank you very much for your feedback on our paper. Your comments were very helpful in improving the quality of the work.

Below you will find an overview of the changes we made compared to the original version.

Kind regard,

Siegfried Müller

Hochschule für Politik München
Institutionell selbstständige Einrichtung
an der Technischen Universität München
Körperschaft des öffentlichen Rechts

Jürgen Pfeffer
Associate Professor (W3)
Richard-Wagner-Straße 4
80333 München

Tel. + 49 089/907793-200
juergen.pfeffer@tum.de
www.pfeffer.at
http://www.hfp.tum.de

HypoVereinsbank München
IBAN: DE67 7002 0270 0006 6162 75
BIC: HYVEDEMMXXX

Reviewer #1: Referee report for PONE-D-22-03432 ‘Reviewing the potentials of MMOGs as research environments’

Reviewers feedback:	Changes made:
While this area of study is certainly needful in light of steep advancements in the development and use of virtual platforms, unaddressed statistically concerns, make it difficult for these findings to be generalized	Based on the feedback, we have identified the following areas needing improvement:<br><br>1. The statistical part of the paper needs to be more transparent and expanded.<br>→ We, therefore, also present the analyses that were performed but could not be employed for various reasons.<br>→ In addition, we have incorporated the feedback received and performed further analyses on this basis.<br><br>2. It needs to be worked out more clearly how Williams’ mapping principle can be applied in the context of our example study and where the limitations of this approach lie (e.g., the availability of data).<br>→ Based on Williams’ framework, we added two additional tables to provide deeper insight into the context and environmental conditions of the game Travian.<br>→ Without these tables, it was factually impossible to see what data (for use as control variables) was available in the Travian dataset being used.<br>→Further, we reorganized and revised this study design section and the statistical tests section for better transparency.
A key area of concern in the literature, and as highlighted by the authors to be the contribution of the current study, relates to the need for a mapping principle and framework, so as to aid the generalizability of results.<br><br>However, the statistical approach of the study does not address this framework, failing to account for the influence associated with the research environment, a key tenet of the	This is indeed a critical point, which we have tried to address as well as possible. We understand that a key aspect of the framework is to allow researchers to compare MMOG research settings. The environmental factors (applied as control variables) play an important role in this. To address this valid concern as best as possible:<br>→ We have reorganized the section and

framework of which the study is primarily based on (see points #13- 15).	introduced an additional table explaining what these environmental conditions look like in the game worlds of Travian.<br>→ Unfortunately, it turned out that the desired data (besides group size) were not available, so it was impossible to include these environmental variables (controls) in the analysis.<br>→ As a positive side effect of this extension, it became clear that many environmental conditions in the world of Travian (e.g., permanence, size of the world, communication medium, etc.) are very close to those in the real world.
Further, while findings from past literature are generally well-integrated, the authors should concise points made by these various sources instead of populating paragraphs with direct quotes. This significantly reduces the readability nor is it entirely appropriate.	We have taken this suggestion and improved readability by changing several direct quotes to indirect quotes.
1) Page 2, line 9<br>"Williams" is not cited here, as it should be.	Corrected
2) Page 2, line 18-21<br>It is not made explicit how unconsciously responding to media relates to the brain having evolved before the existence of media.	This aspect is not relevant to the scope of this paper. Therefore, we removed it.
3) Page 3, line 30-31<br>While the previous examples supposedly illustrates how real-world behaviours may be mapped onto a virtual setting, the introduction of this point about machine learning utility in identifying interactions between network patterns and team performance is abrupt.<br><br>The authors may wish to remove this point or reposition it to a more appropriate location within the introduction.	That is a valid point. Hence, we removed this line.

4) Page 4, line 91,<br>The preliminary introduction of the acronym SNA needs to be first spelled out.	Corrected
5) Page 4, line 116,<br>The citation for “Burt” should be denoted within this sentence.	Corrected
6) Page 5, line 157,<br>The comma after formal networks appear to be redundant.	Removed
7) Page 5 line 171<br>While this line is aimed at introducing readers to the context under which a team operates, the link to “opportunities and constraints” are not immediately apparent. As such, authors may just chose to do without “(e.g., opportunities and constraints)”. The authors may wish to instead include relevant sub-headers between the parentheses, for example, “(i.e., density, transitivity, leadership patterns)”.	That is true. This aspect is not really relevant to the scope of this paper. Therefore, we removed it.
8) Page 8, line 180; page 9, 324; page 9, 354<br>While centrality and k-core are sub-sections under “Leadership Patters in Communication Networks”, the subsequent headings do not suggest this. Changes in the formatting of “Individual Level Centrality”, “K-core” and “Group Level Centrality” headings will improve readability	We have adjusted the headings to a consistent format and also expanded them with additional notes.
9) Page 8, line 289<br>The location of the citation appears to be out of place.	Corrected
10) Page 8, line 296<br>There appears to be a duplicate citation.	Corrected
11) Page 12 ,line 469<br>The authors should justify why Travian was chosen.	In fact, this explanation is missing, and we have integrated this (page 14, lines 542-544 in 'Revised Manuscript with Track Changes').

12) Page 14, line 570-574<br>The authors may wish to align subsequent headers in accordance to the order of the framework explicated here to improve readability. Alternatively, the author may wish to reorder the framework here.	The section has been revised. All points are now stringently ordered (page 16, lines 640-643).
13) Page 18, line 741-744<br>It is mentioned here that items in the framework may be found under the section of “Research Settings (MMOG Travian)” they is no such section heading.	We have fixed this and integrated two additional tables to improve transparency about the context in which the actors operate. The corresponding information can now be found here (page 17, Table 1 & page 18, Table 2).
14) Page 18, line 740-741<br>The authors mentioned the importance of taking into account these contextual factors of the research environment, however, it is not explicated how these contextual factors were statistically accounted for.	That is right and requires further explanation:<br><br>Unfortunately, the contextual factors mentioned in Williams’ framework were mostly unavailable to us. The Travian dataset was collected in 2009/10, and therefore we had to face clear limitations regarding data availability. Because of this lack of available data (secondary dataset), it was impossible for us to incorporate such controls into our analysis.<br><br>To achieve better transparency for the reader, we have integrated the original tables from Williams’ framework (page 17, Table 1 & page 18, Table 2). With the help of these tables, the reader can now see what contextual factors Williams' framework suggests for comparing virtual worlds. In addition, the reader gets a better impression of what this framework looks like in the world of Travian and what data is available for analysis here.
15) Page 19, line 754-757<br>Given that factors relating to the research	That is, in fact, a critical point and also

environment should be properly accounted for, it is unclear how Spearman's rank-order correlation would be the best suited option, even with respect to concerns of the strong correlation between independent variables and non-normal distribution.<br><br>To explicate, it is not clear if the independent variables necessarily have to be examined concurrently with respect to team performance. In other words, why not transforming the data and using a linear regression for each independent variable? Or instead, why not structural equation modelling and allowing the independent variables to covary? Both these statistical methods allow for the effects of control variables to be accounted for.	requires further explanation from our side.<br><br>In the last version of our paper, we failed to make the reader aware that we are not able (due to a lack of data) to follow Williams' approach in an ideal way.<br><br>In particular, it was not previously apparent that such context variables were not available as controls.<br><br>The extensions (two tables and comments) we have now built in show that, in effect, we only have one control variable (group size) available.<br><br>Our further considerations, built on this:<br><br>After splitting our data set into three subsamples (small, medium, and large), resulting in a set of comparable groups, the effect of group size on team performance almost disappeared. Thus, we were faced with the decision of whether to include the only control variable (group size) in our analysis (regression approach) or to go for a correlation technique in favor of other advantages (here: ordinal performance data, Spearman).<br><br>We calculated and evaluated both approaches. This revealed that the results of both approaches were very similar. We have attached the table of results of the regression analysis, demonstrating the very weak relationship between team size and team performance (at a low level of significance).<br><br>In addition to these two alternatives, we computed traditional multiple regression models and included them for transparency as well. Due to the high degree of collinearity (VIF scores), these were not usable for our analysis. Similarly, structural

	equation models were not usable for our purposes due to the fact that the theoretical foundations (models) of how the interaction of the individual network measures could look still do not exist. We believe that the use of structural equation modeling is very promising for developing and testing hypotheses about how these structural network patterns interact, and we plan to address this topic in future work.
16) Page 21, line 830-835<br>The authors initially appropriately highlighted that while mapping was successful in their study, this may not translate to all virtual environments (line 830-833). However, the authors proceed to make the conclusion that it might not be important to distinguish between online and offline worlds, directly contradicting the previous statement.	The contradictory sentence has been removed.

Reviewer #2: Referee report for PONE-D-22-03432 ‘Reviewing the potentials of MMOGs as research environments’

Reviewers feedback - major comments:	Changes made:
1. I was not particularly convinced that major contribution is showing that MMOG networks are just like real-work networks. Is that of substantive importance? The paper could just as easily be presented as an application of social network techniques to the behaviour of leadership/organisational behaviour in MMOGs, based on a substantive data example. Is that the patterns found loosely resemble those found in real-world networks simply to do with that involvement of human actors and the stakes/context their interactions take place in?	When I started my Ph.D. project on network structures in MMOGs, I talked to a couple of leading researchers in this area. Most of them told me that future research in this area is very promising (mainly because of the huge amounts of data) but that they had major concerns about the results of this research being transferable to the real world. To date, this skepticism has dominated the field of network-based team and leadership research, and the few papers that exist in the field have not been able to draw a clear conclusion.<br><br>With my current example, I want to show that (1) there are some MMOG worlds that can represent real-world working environments and (2) that many different types of MMOGs exist, and therefore one cannot make a general judgment.<br><br>This second point became especially clear to us during the current revision, which is why we have revised the “Study Design” part once again. Among other things, we went deeper into Williams’ framework here and added two additional overview tables with the characteristics of the game Travian.
2. The paper is clearly written but does feel repetitive. Could the definitions of centrality, k-core, etc., which appear on pages 8-10 and again 15-16 be merged? I personally would have preferred the concepts to be introduce alongside the descriptions of the statistics, because I found the concepts to be hard to follow in the abstract without any graphical schematic or equation to follow (especially k-	This is an understandable point. We have followed this suggestion and merged the passages on pages 8-10 and 15-16.<br><br>In this way, the theoretical descriptions of each concept are now found in direct proximity to the formal descriptions (equations).

core: what is a maximal cohesive subgroup, the hierarchical structure of the k and k+1 cores, etc.). Doing this would also reduce the sense of repetition and prevent readers from forgetting too many details from earlier.	We hope that this improves readability.
3. Statistical analysis: I am a little surprised that multicollinearity prevented a regression analysis from being performed. The sample sizes are small but not too small. We are thus prevented from understanding the partial effects of each network measure (i.e. those of a per-unit increase with the other measures held fixed) on the performance outcome, and must rely on bivariate effects instead. Non-normality could have been dealt with using a nonparametric bootstrap of one form or another. I would ideally like to see the regression analysis results or, at least, more justification as to why a regression analysis was not performed, because the reasoning currently given (pages 18-19) are vague.	This is an important point. Indeed, we should have described why we discarded certain approaches better. Therefore, we have revised this section and integrated additional (background) information.<br><br>Now we have explained the results of the regression analysis we performed. In fact, the sample size and non-normality of the data did not present insurmountable hurdles. We were able to perform multiple regression analysis without difficulty using the bootstrap procedure. However, the multicollinearity, which is reflected in the high VIF values, proved to be problematic and prevented the use of the multiple linear regression method in the end. The detailed analyses can be found attached.
Reviewers feedback - minor comments:	Changes made:
4. From page 5: I would like a clearer, if brief, statement about the importance of communication and support for the study of leadership and performance. The importance emerges the more one reads (especially on page 14), but it would be helpful to flag the key reasons earlier.	To help the reader to understand better why we chose these two network types, we have added a short passage to this section (page 5, lines 156-166 in 'RevisedManuscript with Track Changes'). The focus here is to highlight the central role of communication in team interaction. In addition, we discuss whether support actions are a good indicator of the extent to which a team can coordinate.
5. Page 12, discussion of alliances. A simple worked example linked to a graphical representation would help the reader no end in understanding how these alliances are formed and how each works together and its overall performance measured.	The way teams are formed in Travian is a very hierarchical one. A key element of this is that the leader invites the members and subsequently gives them the legitimacy (power) to perform their roles.

	To illustrate this, we have added an additional figure to the section (page 15, Figure 1).
6. Table 1: To clarify, there are 352 teams (of small, medium and large sizes) and the total number of individuals in these teams is 1179, but each person is in one team? Or is it that there are 352 teams and 1179 – 252 single-person teams? If the former, this needs to be made clearer in the table because it is confusing to mix sums of different unit sizes.	Yes. This is correct. There are 352 teams (of small, medium, and large sizes). In addition, the total sample of teams (of all group sizes) consists of 1,179 teams.<br><br>To avoid any misunderstanding here, we have made the labeling of the groups more detailed (page 16, lines 676-678). Additionally, the delineation of the different groups can be seen in Figure 3.
