# Supplementary material for: Reviewing the potentials of MMOGs as research environments: A case study from the strategy game Travian
Source: PLoS One. 2023 Feb 15;18(2):e0281114. doi: 10.1371/journal.pone.0281114 (PMC9931136; doi:10.1371/journal.pone.0281114)
Supplement: Attachment — Submitted filename: Response to Reviewers.pdf [file pone.0281114.s004.pdf]

Bavarian School of Public Policy
Technical University of Munich

Hochschule für Politik München | Richard-Wagner-Straße 1 | 80333 München
Professor of Computational Social Science & Big Data

Munich, January 2, 2023

Journal PLOS ONE
Submission: Revised research article

Response to Editor

Dear Keith Leverett Warren,

Thank you very much for your final feedback on our paper. Your additional comments are appreciated and are helpful in improving the quality of our work.

Below you will find an overview of the changes we made compared to the previous version.

Kind regard,

Siegfried Müller

Editors feedback:	Changes made:
First, in your materials and methods section you state that the maximum team size is 60, but the number of individuals in a team in your dataset goes up to 145. The discrepancy, which may be due to the fact that members can leave alliances and be replaced by new members, needs to be explained. (If you did explain it and I missed it, please point me to where you explained it.)	This is a valid point. To address this, we have made an enhancement in the study design section at the group size subheading. We now also address the fact that, in addition to game design, the choice of aggregation periods also matters and influences the quality of the dataset. So far, the reader had to deduce this for himself.
Second, in your results section you state that higher betweenness leads to lower performance for medium and large teams. But your table indicates that these relationships are nonsignificant.	We have taken up this point. In the relevant passages, we have therefore again clearly pointed out that the results can at best be regarded as indications due to the lack of significance. Further, we have adjusted the related part in the paper's abstract.
