# Supplementary material for: Reviewing the potentials of MMOGs as research environments: A case study from the strategy game Travian
Source: PLoS One. 2023 Feb 15;18(2):e0281114. doi: 10.1371/journal.pone.0281114 (PMC9931136; doi:10.1371/journal.pone.0281114)
Supplement: Attachment — Submitted filename: Referee report for PONE.pdf [file pone.0281114.s002.pdf]

Referee report for PONE-D-22-03432 'Reviewing the potentials of MMOGs as research environments'

This clearly written paper uses data obtained from the Travian MMOG to test research hypotheses based on the findings of real-world studies of organisational effectiveness and leadership. These hypotheses boil down to testable associations between measures of organisational performance and measures which capture the structure of complex networks. The authors provide a comprehensive review of the real-world literature on network organisation, an explanation of the statistics used to measure network structure, an analysis based on the Travian data obtained by the authors, and a discussion of the results. Bar the points below, I feel this work potentially makes a valuable contribution.

Major comments

1. I was not particularly convinced that major contribution is showing that MMOG networks are just like real-work networks. Is that of substantive importance? The paper could just as easily be presented as an application of social network techniques to the behaviour of leadership/organisational behaviour in MMOGs, based on a substantive data example. Is that the patterns found loosely resemble those found in real-world networks simply to do with that involvement of human actors and the stakes/context their interactions take place in?
2. The paper is clearly written but does feel repetitive. Could the definitions of centrality, k-core, etc., which appear on pages 8-10 and again 15-16 be merged? I personally would have preferred the concepts to be introduce alongside the descriptions of the statistics, because I found the concepts to be hard to follow in the abstract without any graphical schematic or equation to follow (especially k-core: what is a maximal cohesive subgroup, the hierarchical structure of the k and k+1 cores, etc.). Doing this would also reduce the sense of repetition and prevent readers from forgetting too many details from earlier.
3. Statistical analysis: I am a little surprised that multicollinearity prevented a regression analysis from being performed. The sample sizes are small but not too small. We are thus prevented from understanding the partial effects of each network measure (i.e. those of a per-unit increase with the other measures held fixed) on the performance outcome, and must rely on bivariate effects instead. Non-normality could have been dealt with using a nonparametric bootstrap of one form or another. I would ideally like to see the regression analysis results or, at least, more justification as to why a regression analysis was not performed, because the reasoning currently given (pages 18-19) are vague.

Minor comments

4. From page 5: I would like a clearer, if brief, statement about the importance of communication and support for the study of leadership and performance. The importance emerges the more one reads (especiallyon page 14), but it would be helpful to flag the key reasons earlier.
5. Page 12, discussion of alliances. A simple worked example linked to a graphical representation would help the reader no end in understanding how these alliances are formed and how each works together and its overall performance measured.
6. Table 1: To clarify, there are 352 teams (of small, medium and large sizes) and the total number of *individuals* in these teams is 1179, but each person is in one team? Or is it that

there are 352 teams and 1179 – 252 single-person teams? If the former, this needs to be made clearer in the table because it is confusing to mix sums of different unit sizes.
